# Attenuated glutamate induced ROS production by antioxidative compounds in neural cell lines

**DOI:** 10.1039/c9ra03848e

**Published:** 2019-10-28

**Authors:** Haolin Xin, Ying Cui, Zhongping An, Qian Yang, Xuan Zou, Ning Yu

**Affiliations:** Tianjin Key Laboratory of Cerebral Vascular and Neurodegenerative Diseases, Department of Neurology, Nankai University, Huanhu Hospital Tianjin China uyning@163.com; Tianjin University of Traditional Chinese Medicine Tianjin China

## Abstract

Glutamate is an excitatory neurotransmitter involved in neural function. Excess accumulation of intercellular glutamate leads to increasing concentration of reactive oxygen species (ROS) and reactive nitrogen species (RNS) in neuronal cells. In this study, we investigated the antioxidant activity of several typical superior compounds among four neuronal cells, and determined the scavenging activity of free radicals. The *in vivo* assay was also carried out to compare the protective effect of glutamate-induced cell damage. Hierarchical clustering analysis was used to identify the common properties. Glutamate induced neurotoxicity and ROS production, suggesting glutamate cytotoxicity was related to oxidative stress and widely exists in different cell lines. Those screening compounds exhibited strong antioxidant ability, but low cytotoxicity to neuronal cells, acting as agents against neurodegenerative diseases. Finally, a hierarchical clustering analysis assay indicated that hyperoside and rutin hydrate are the most effective compounds for attenuating intercellular ROS levels. The results suggested the activity more or less relies on structure, rather than residues. These data generate new supporting ideas to remove intracellular ROS and the identified compounds serve as potential therapeutic agents in multiple neurological diseases.

## Abstract 2

Glutamate is an excitatory neurotransmitter involved in neural function.

## Graphical abstract

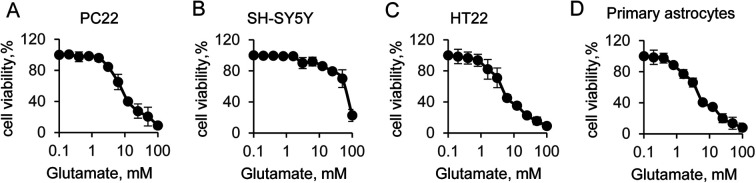

## 1. Introduction

Glutamate, an inhibitory and excitatory neurotransmitter in the central nervous system (CNS), plays an essential role on regulating brain function, and contributes excitotoxicity in neuronal cells.^1–3^ Excessive stimulation of intercellular glutamate is a leading factor, which is involved in neurodegenerative diseases of the CNS, including strokes, Alzheimer's disease and Parkinson's disease.^4,5^ Glutamate acts as a neurotoxicant mainly by increasing the intracellular levels of reactive oxygen species (ROS), as well as reactive nitrogen species (RNS) in neuronal cells.^6^ There is accumulating evidence, supporting the fact that oxidative stress is a causal factor related to neurodegenerative disorders.^7^ Thus, glutamate induced neuronal injury, through the formation of ROS has been widely proven.^6^ The additional glutamate over physiological level induced oxidative stress, which leads to neuron apoptosis and necrosis. In addition, oxidative stress can trigger the protease cascade events, results in protein misfolding, mitochondrial dysfunction. In those process, glutamate-induced neurodegenerative diseases and neuronal cell death is usually associated with oxidative stress.^8–10^ The over accumulated ROS, as well as the oxidative stress, are tightly associated with inhibition of glutathione synthesis, nitrosative stress *et al.*^11–13^ Several antioxidants compounds has been investigated to reduce oxidative stress, and potentially served as therapeutic agent.^14–17^ Polyphenols are one of the groups exhibit strong free radicals scavenging activity. Therefore, multiple typical compounds, harboring different polyphenol content and molecular weight were selected (Fig. 3A).

Antioxidant compounds are usually natural substances, perform effective anti-cancer, anti-inflammatory, anti-proliferative and anti-neurodegenerative disease ability.^18–20^ Phenolic and polyphenolic compounds are the primary antioxidants, which reveal a linear relationship between the total phenolic content and the antioxidant properties.^21^ The antioxidant activity that eliminates intercellular ROS has been widely studied, including the prevention of neuronal dysfunction and neural cell death.^22–24^ Oxidative stress is an imbalance between free radical production and antioxidant defenses in CNS system.^25^ Those compounds share common polyphenols residues in their chemical structure, which is critical for attenuating ROS production. However, they might differently target inside the cells, due to their unique structure and larger molecular size.^26,27^ Therefore, it is worth to try hierarchical clustering analysis based on activity of those compounds.^28^

Here, we explored the antioxidant effect of various compound and compared glutamate-induced neurotoxicity among neuronal cells. Indeed, glutamate contributes excitotoxicity in all tested neuronal cells. In addition, it is tightly associated with intercellular ROS production when subject to expose with the half maximal inhibitory concentration (IC_50_) of glutamate. We utilize superior antioxidant activity compounds and carried out antioxidant effect assay *in vivo* and *in vitro*. When the cells were supplied with any superior antioxidant compound, it exhibit effective radical scavenging activity, however the cell viability was not affected. Those compounds partially attenuate the ROS and RNS production, and were further grouped by hierarchical clustering analysis. Those results indicated that hyperoside and rutin hydrate are clustered as the most effective group. The two compounds, exploring larger molecular weight than other tested ones, decreased glutamate-induced ROS production. Those compounds support antioxidant activity, as well as neuroprotective effect against glutamate. The results implied the protective effect was predominantly rely on both chemical residues and structure. Notably, the primary astrocytes was less susceptible than other cell lines, and grouped separately in hierarchical clustering assay. Overall, these results outline the antioxidant effect of investigated compounds that protect glutamate-induced ROS production in various neural cells.

## 2. Materials and methods

### 2.1. Materials

Dulbecco's Modified Eagle's Medium (DMEM) (ATCC 30-2002) and Fetal Bovine Serum (FBS) (ATCC 30-2020) were purchased from American Type Culture Collection (ATCC). The cell lines, including rat adrenal medulla pheochromocytoma PC12 and human brain neuroblastoma SH-SY5Y were purchased from ATCC. HT-22, mouse hippocampal neuronal cell line was purchased from Thermo Fisher Scientific. Astrocytes were isolated from the cortices of one-day new born Sprague-Dawley rats as described previously.^29^ Briefly, removing the meninges carefully, digested the cerebral cortices with dispase, then astrocytes were recovered by a 40 μm cell strainer and plated with a density of 1 × 10^5^ cells per ml in DMEM medium.

Cell Counting Assay Kit-8 (CCK-8) was purchased from Gold Biotechnology, China. Trypan Blue Staining Solution was bought from Abcam, USA. The 2,7-dichlorofluorescein diacetate (DCFDA) fluorescence dye was purchased from molecular probe. Total Antioxidant Capacity-Peroxyl Radical Assay Kit (Northwest Life Science Specialties, NWK-TAC01) and OxiSelect™ Hydroxyl Radical Antioxidant Capacity Activity Assay kit (Cell Biolabs, Inc., STA-346) were used. All the antioxidative chemical compounds were purchased from Sigma or Fluka, USA. All other chemicals and regents made in China were analytical grade.

### 2.2. Cell culture and cell viability assay

All the cells were cultured in DMEM medium at 37 °C in a humidified 5% CO_2_ incubator. Unless indicated, 10% (v/v) FBS, 100 U per ml penicillin and 100 μg ml^−1^ streptomycin were added in medium. Replacing with fresh medium every two or three days to maintain regular cell growth. Cell viability was performed using the CCK-8 kit according to manufacturer's instructions. Briefly, 1 × 10^4^ cells were seeded into 96-well plates with the initial volume of 100 μl for each well. After incubation for 24 hours, 10 or 50 μg ml^−1^ catechins and theaflavins, 5 μM glutamate and 50 μM trolox were added as indicated. Cells was incubated with additional 10 μl CCK-8 solution for additional three hours at 37 °C. Finally, the absorbance at 450 nm in each well was measured using Microplate Reader (TECAN 10M).

### 2.3. Flow cytometry analysis of ROS production

The ROS production was evaluated using 2,7-dichlorofluorescein diacetate (DCFDA) fluorescence dye as described.^30^ The cells were normally maintained, when appropriate, cells were treated with 1 μM DCFDA and incubated at 37 °C for 30 minutes in dark. After staining, the cells were passed through a 40 μm cell strainer to get rid of cell clumps. All the cells were then subjected to flow cytometry (FACS Calibar; BectonDickinson), and at least 1 × 10^4^ cells were quantified by FACS equipped with the Cell Quest software.^31^

### 2.4. Antioxidant activity of various compounds

Spectrometric techniques were relied to determine the reaction of antioxidant activity.^32,33^ Briefly, DPPH (2,2-diphenyl-1-picrylhydrazyl),^32^ and ABTS (2,2′-azino-bis-(3-ethylbenzothiazoline-6-sulphonic acid)) assay was used.^33^ The delocalization on the DPPH molecule was determined by the occurrence of purple color, which has an absorbtion with a maximum wavelength around 520 nm. DPPH solution was fresh prepared and in methanol at 0.1 mM. All the compounds were dissolved in methanol and performed a serial dilution once needed. A final volume of 4 ml of DPPH stocking solution was mixed with individual compound. The mixture was rotated in dark at room temperature for 30 minutes. The control samples with DPPH only was included. After rotation, the absorbance at 520 nm was determined. The percent quenching of DPPH was calculated by the following formula: % DPPH scavenging = [(*A*_control_ − *A*_sample_) × 100/*A*_control_].

The ABTS cation radical which absorbs at 734 nm generate a bluish-green color. ABTS stock solution was prepared in water at final concentration 7 mM. Additional potassium persulfate was also supplied to ABTS stock solution at 2.45 mM. The mixture was kept in dark environment at room temperature overnight prior to use. The ABTS radical solution was diluted by ethanol with an absorbance about 0.70 at 734 nm to make the working solution. 1 ml of the working solution was added to 10 ml sample, mixed gently by shaking 5 min. The inhibition was calculated as shown in the following equation: % ABTS scavenging = [(*A*_control_ − *A*_sample_) × 100/*A*_control_].

Peroxyl radical antioxidant capacity was detected by the Total Antioxidant Capacity-Peroxyl Radical Assay Kit. The peroxyl radicals in the kit are generated by thermal decomposition of 2,2′-azobis-(2-amidinopropane) (ABAP). Those peroxyl radicals form a luminol radical by reacting with an indicator molecule, luminol. Finally, it produce an emission light quantified at 425 nm. Once antioxidants are mixed in the assay, luminescence is inhibited. A water-soluble vitamin E (tocopherol) analog, trolox was used as standard to compare with the other tested compounds. A standard curve is generated using trolox as described in the protocol provided by the manufacture. The incubation times for all tested compounds were less than 20 min. The concentration of tested compounds were properly diluted in order to compare with standard curve. All the assay was performed at room temperature.

Hydroxyl radical antioxidant capacity was determined by OxiSelect™ Hydroxyl Radical Antioxidant Capacity Activity Assay kit. Briefly, the hydroxyl radicals produced initially by the kit, quenches the fluorescent probe. Once antioxidants are present, they block oxidation of the fluorescent probe. A trolox standard curve was generated according to the protocol of the kit. The antioxidant capacity of tested compounds correlates to the fluorescence decay curve is employed to compare the hydroxyl radical antioxidant activity. All the assay was performed at room temperature. Reactive nitrogen species (RNS) was also measured, which is another type reactive species. To this end, nitric oxide (NO)-scavenging activity was investigated as described before.^33^ The screening compounds at 64 μg ml^−1^ were individually incubated with sodium nitroprusside (SNP) in 0.1 M phosphate buffer at pH 7.4. In this assay, SNP was used as the initial source of NO by light irradiation was necessary for release of NO. The compound and SNP mixture was incubated at room temperature, where a SNP only reaction was used as control. The nitrite level was determined after incubating 30 min, than Greiss reagent system, harboring 1% sulphanilamide and 0.1% naphthylethylenediamine in 5% phosphoric acid was used. The reaction mixture was finally examined by the absorbance at 546 nm.

### 2.5. Hierarchical clustering analysis and statistical analysis

Hierarchical clustering analysis (HCA) was done using Biovinci software. The relative decreased potation of ROS production in individual cell group was employed. Statistics was performed using Euclidean distances between decreases of the ROS production.^34^ The result was visualized using heat maps.

All the data was presented as average ± standard deviation (SD). At least three biological repeats were used unless otherwise indicated. The student's *t*-test was used to evaluate significant between changes, where *P* < 0.05 is considered significant (*).

## 3. Results

### 3.1. Glutamate induced cell viability assay on various neural cells

**Fig. 1:**
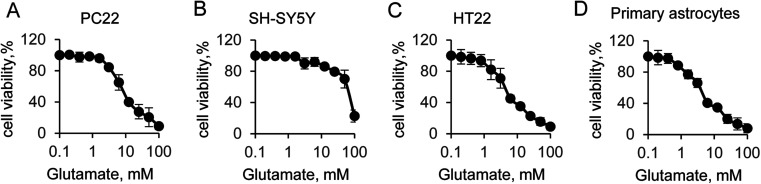
Glutamate-induced neural cell viability. (A) PC22, (B) SH-SY5Y, (C) HT-22 and (D) primary astrocytes cell viability by CCK-8 assay.

Glutamate-induced cytotoxicity is one of the leading causes of neuronal cell death.^35^ In this study, we first compared the cytotoxicity among different neural cells. The cell viability experiment was performed to four typical neural cells, including PC22, SH-SY5Y, HT22 and primary astrocytes. The initial concentration of glutamate was 100 mM, and followed by a two-fold serial dilution. Incubation with glutamate in the medium for 12 hours, results in significantly decrease the cells viability in a does dependent manner, which indicated the toxic effect to all tested cell lines (Fig. 1). Indeed, a strong cytotoxicity was found when supplied with 100 mM of glutamate for all tested cells. Notably, additional 100 mM glutamate to SH-SY5Y cells induced a sharp decrease of the cell viability, suggesting that SH-SY5Y was more resistant to glutamate than other cells. However, when supplied with lower concentration of glutamate, which was less than 25 mM, the cells viability was almost identical to control cells. The half maximal inhibitory concentration (IC_50_) of glutamate for all cells were then quantified by solver function in excel. The IC_50_ of glutamate induced cytotoxicity for 12 hours was 8.8 mM, 67 mM, 6.9 mM and 5.1 mM for PC22, SH-SY5Y, HT22 and primary astrocyte, respectively. Next, the value that close to the IC_50_ was used to the following experiment. These results suggested glutamate induced neurotoxicity was widely exist in different cell lines, and the SH-SY5Y cells was more susceptible.

### 3.2. Glutamate induced ROS production on various neural cells

**Fig. 2:**
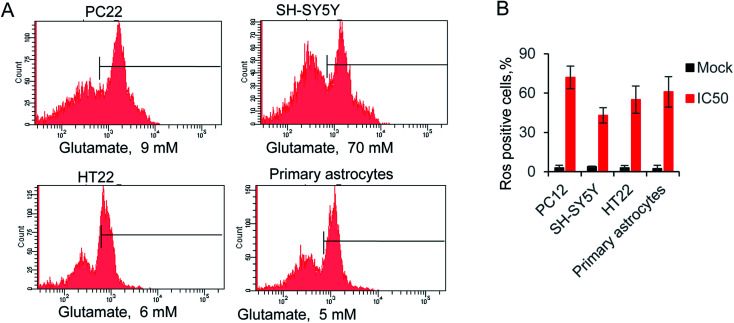
(A) Glutamate-induced ROS production in neural cells, using ROS-sensitive fluorometric probe DCFDA by flow cytometry (*n* = 10 000 cells). (B) Relative ROS production in all tested cells.

The reactive oxygen species (ROS) triggered by glutamate was identify as a major reason for cells death.^36^ Hence, we evaluated the relative ROS production by supplying with IC_50_ concentration glutamate for each cell. The accumulation of ROS was measured by a typical way, utilizing a converting reaction of DCFH-DA to DCF.^37^ Glutamate treatment significantly increased the ROS level to 72.1% 42.7%, 55.4% and 61.4% in four kinds of cells, whereas ROS production in the control groups were all lower than 4% (Fig. 2A and B). Notably, the glutamate used in SH-SY5Y is 70 mM, due to the high level of IC_50_ in this cell line. The result exhibited only 42.7% of ROS positive cells, which is lower than the other groups. The results indicated the neuromodulator glutamate, through specific substrate interactions, enhances intercellular ROS production in neural cells.

### 3.3. Attenuated glutamate excitotoxicity by antioxidant compounds

**Fig. 3:**
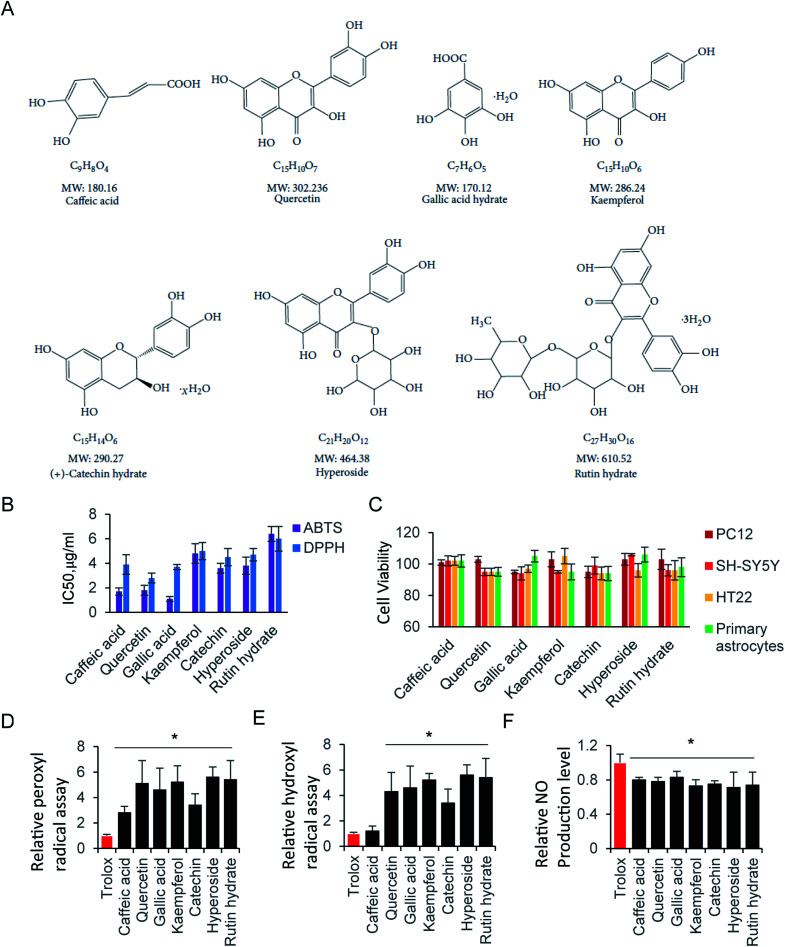
Radical scavenging activity of antioxidant compounds. (A) Chemical structure of selected compounds. (B) IC_50_ of ABTS and DPPH assay. (C) Cell viability assay at 64 μg ml^−1^ compound in various cells. Relative (D) peroxyl radical and (E) hydroxyl radical assay. (F) NO scavenging activity assay.

ROS, originate from oxygen, was naturally produced as part of the metabolism within the cytoplasm. Excess ROS have a fatal effect on intercellular toxicity and dysfunction. The potential antioxidant activity of the commonly compounds has been widely investigated. Here, we selected several typical superior antioxidant activity compounds, and their chemical structure is shown as in Fig. 3A.^38^ The antioxidant activity of those compounds was observed by 2,2′-azinobis-(3-ethylbenzothiazoline-6-sulfonic acid) radical cation (ABTS^+^) decolourisation assay. The results showed that inhibition of ABTS^+^ by different compounds were in a concentration dependent manner (Table 1). Among all the tested compounds, caffeic acid, quercetin and gallic acid showed the best ability of scavenging ABTS^+^ (95.2%, 94.2% and 99.2% at 64 μg ml^−1^), and accordingly, catechin (92.8% at 64 μg ml^−1^) inhibited ABTS^+^ more than kaempferol, hyperoside, and rutin hydrate (83.3%, 83.5% and 88.2% at 64 μg ml^−1^). Next, we determiner the scavenging ability of the same compounds on DPPH as (Table 2). The results presented the decrease in absorbance of the DPPH radical due to the scavenging abilities of the extracts of different compound. The data exhibited the similar inhibiting abilities of DPPH to that of ABTS^+^. The IC_50_ of ABTS^+^ and DPPH were further calculated and shown in Fig. 3B. The compounds with small molecular weight exhibit relative lower IC_50_, and it is probably because of the high content of polyphenols.

**Table 1:** ABTS scavenging activity at different concentrations^a^

Concentration μg ml^−1^	Caffeic acid	Quercetin	Gallic acid	Kaempferol	Catechin	Hyperoside	Rutin hydrate
1	39.8 ± 1.7	35.8 ± 4.3	48.8 ± 4.7	33.4 ± 5.2	27.8 ± 1.3	43.1 ± 4.2	23.5 ± 8.5
4	71.4 ± 5.1	70.4 ± 5.3	80.4 ± 5.3	47.5 ± 6.5	64.4 ± 3.1	58.1 ± 5.2	37.1 ± 2.4
16	88.3 ± 6.9	85.9 ± 6.4	95.9 ± 2.4	63.2 ± 3.7	78.8 ± 2.9	68.2 ± 2.5	74.5 ± 4.6
64	95.2 ± 5.6	94.2 ± 4.2	99.2 ± 1.2	83.3 ± 4.1	92.8 ± 5.5	83.5 ± 2.6	88.2 ± 3.4

a Data are expressed as mean ± SD of *n* = 6.

**Table 2:** DPPH scavenging activity at different concentrations^a^

Concentration μg ml^−1^	Caffeic acid	Quercetin	Gallic acid	Kaempferol	Catechin	Hyperoside	Rutin hydrate
1	29.2 ± 5.7	38.8 ± 7.8	44.3 ± 5.7	30.4 ± 7.1	29.8 ± 7.3	42.1 ± 5.5	28.5 ± 5.5
4	51.2 ± 5.4	75.4 ± 3.2	75.4 ± 4.3	42.1 ± 2.5	45.4 ± 8.7	56.1 ± 6.2	36.1 ± 4.4
16	73.3 ± 2.3	86.9 ± 6.3	93.5 ± 3.4	61.2 ± 3.7	72.8 ± 5.9	68.2 ± 6.6	78.5 ± 4.7
64	85.4 ± 5.3	96.2 ± 4.1	98.2 ± 0.2	80.3 ± 4.4	85.5 ± 8.3	86.5 ± 6.3	89.2 ± 4.4

a Data are expressed as mean ± SD of *n* = 6.

Although the radical scavenging activity of those compounds were tested by ABTS^+^ and DPPH assay. They radicals are not present in human body. Consequently, peroxyl radical and hydroxyl radical antioxidant capacity were performed, where trolox was used as the standard to compare with tested compounds. There is no significant difference of peroxyl radicals antioxidant capacity between trolox and caffeic acid. Other than that, all compounds exhibit stronger antioxidant ability of peroxyl and hydroxyl radicals than trolox (Fig. 3D and E).

The RNS, another type reactive species, is also contributes significantly to oxidative cell damage. Therefore, it might be beneficial if screening compounds could potentially scavenge NO. Indeed, all tested compounds inhibited the NO production. They exhibited similar scavenging NO ability, with the relative NO production level at 70–80% compared with control (Fig. 3F). Here, the NO scavenging activity may be not relative with the molecule weight, but relay on the residue of hydroxyl of those compounds. Next, we evaluated the cell viability of those compounds at 64 μg ml^−1^ by using CCK-8 Assay. As shown in Fig. 3C, all tested compounds did not affect cell viability at 64 μg ml^−1^, indicating that those compounds were not toxic to neural cells. In addition, the trypan blue exclusion test was performed to all tested compounds at 64 μg ml^−1^. There is a clear cytoplasm when compound was added in cells for 24 hours, indicating the cells are viable for all those compounds. Taken together, those compounds exhibited strong antioxidative ability, and low cytotoxicity to neural cells.

### 3.4. Attenuated glutamate induced ROS production by antioxidant compounds

**Fig. 4:**
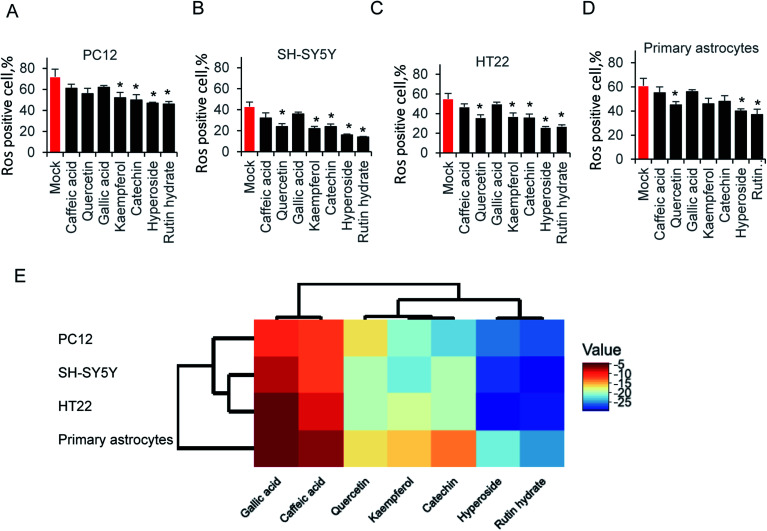
Attenuated glutamate-induced ROS production of (A) PC22, (B) SH-SY5Y, (C) HT-22 and (D) primary astrocytes ROS production by flow cytometry. (E) Hierarchical clustering analysis of ROS production.

Exposure of neural cells to glutamate, leads to ROS production. To elucidate the ability of those compounds on attenuating glutamate-induced oxidative stress, cells were pretreated with 64 μg ml^−1^ compounds for 12 hours before glutamate induced ROS production. As shown in Fig. 4A, kaempferol, catechin, hyperoside and rutin hydrate attenuated ROS production in PC12 cell. Accordingly, we examined SH-SY5Y cells in Fig. 4B, the results is similar as PC12. One more compound, catechin could also decrease the ROS production. In addition, we tested HT22 and primary astrocytes in Fig. 4C and D. Indeed, the same pattern was found as shown in HT22 cell. These results indicated that only partial sets of tested compounds attenuated ROS production at indicated concentration. Furthermore, different types of compounds were grouped by hierarchical clustering analysis, as shown in Fig. 4E. The difference of ROS positive cells between mock and compound treatment groups was used to generate hierarchical clustering analysis. The gallic acid and caffeic acid pretreated, results in small decrease of ROS positive cells, which is usually less than 10%. Quercetin, kaempferol and catechin share relatively close distance, and play a moderate role on ROS production. While, hyperoside and rutin hydrate are the strongest compounds on attenuating glutamate induced ROS positive cells. These results suggest that those antioxidant compound could reduce glutamate-induced ROS production.

## 4. Discussion

The antiradical activity could be evaluated by different assays and results strongly depended on the pure compounds or plant extracts in those assays.^39^ The chemiluminescent luminol assay is usually more sensitive than the colorimetric assays. One the other hand, some antiradical compounds in plant extracts mixture might affect the determination of antiradical activity. Therefore, the DPPH and ABTS assays results usually varies, and also strongly depended on environment conditions.^40^ The antioxidant activities of the reference antioxidants and selected polyphenol compounds might vary in different assays. Indeed, it is tightly determined by different specificities of solvents, reagents, molecular size and the functional groups of the compounds.^41^ The study provide a remarkable comparison of the antioxidant chemical compounds. The ABTS and DPPH radical-scavenging activity, peroxyl and hydroxyl radical antioxidant capacity assay indicated strong antioxidant ability in all tested compounds. Here, the IC_50_ rate range from 2 μg ml^−1^ to 6 μg ml^−1^ in ABTS and DPPH assay. On the other hand, anti-oxidative stress of those polyphenols in neural cell are different. Hyperoside and rutin hydrate, strongly attenuated glutamate induced ROS production. This study provides evidence that hyperoside and rutin hydrate can also exert a neuroprotective effect on glutamate excitotoxicity.

Hyperoside and rutin hydrate are usually present plants including citrus fruit. Those compounds, which share larger molecular weight than other tested ones, usually has low bioavailability due to poor absorption, but high metabolism.^42,43^ However, those two compounds exhibited ability reducing ROS production in various neural cells, which is shown as hierarchical clustering analysis in Fig. 4E. In particular, their influence on glutamate induced ROS production toward their regulatory function in view of very low concentrations needed for the activity than other tested compounds in this study. The effect of on cellular signaling is well described which interact with many proteins of signaling cascades.^44–46^ Here, it is possible that those compounds may be responsible for mediating ROS-induced signaling cascades.

Glutamate excitotoxicity, one of the leading inducer to neuronal cell death, produce oxidative stress in most cases.^47–49^ Indeed, our data showed that glutamate strongly induced ROS production at individual IC_50_ concentration judged by CCK-8 cell viability assay. The antioxidant ability in cell free system is mainly due to the polyphenol residues, which is tested by ABTS and DPPH assay in Tables 1 and 2, as well as Fig. 3B. All those seven compounds generate radical scavenging affect, while behaved differently to restore glutamate induced ROS production (Fig. 4). The neuroprotective effect against glutamate-induced cell damage, especially ROS production and its secondary effect was not due to the partial polyphenol residues, which is exist in all compounds. It is probably mediated through attenuation of oxidative stress enzymes.

## 5. Conclusion

Glutamate cytotoxicity was related with oxidative stress and widely exist in neuronal cells. The screening compounds exhibit the protective function on glutamate-induced neuronal cell damage, both in subculture and primary culture cells. They ubiquitously effective on ROS and RNS scavenging ability *in vitro*, indicating the diverse radicals substrates. Those polyphenol compounds have strong antioxidant ability, but relative low cytotoxicity. The compounds, harboring large molecular weight, hyperoside and rutin hydrate, are the most effective ones that attenuated intercellular ROS level. These data outline a new strategy on glutamate induced CNS degradation disease, expand the candidates for potential therapeutic agent.

## Author contributions

Haolin Xin, Zhongping An, Qian Yang, Xuan Zou performed the experiment. Haolin Xin, Ying Cui and Ning Yu analyzed the data and wrote the manuscript.

## Conflicts of interest

The authors declare no conflicts of interest.

## Supplementary Material
